# Diagnostic and Prognostic Roles of Thrombospondin-2 in Digestive System Cancers

**DOI:** 10.1155/2022/3749306

**Published:** 2022-07-14

**Authors:** Feiqiong Gao, Wenyi Chen, Tingxiao Zhao, Jiong Yu, Xudong Feng, Lan Wang, Tianan Jiang, Hongcui Cao

**Affiliations:** ^1^State Key Laboratory for the Diagnosis and Treatment of Infectious Diseases, Collaborative Innovation Center for Diagnosis and Treatment of Infectious Diseases, The First Affiliated Hospital, Zhejiang University School of Medicine, 79 Qingchun Rd., Hangzhou City 310003, China; ^2^National Clinical Research Center for Infectious Diseases, Hangzhou, China; ^3^Key Laboratory of Diagnosis and Treatment of Aging and Physic-Chemical Injury Diseases of Zhejiang Province, 79 Qingchun Rd, Hangzhou City 310003, China; ^4^Department of Ultrasound, The First Affiliated Hospital, Zhejiang University School of Medicine, 79 Qingchun Rd., Hangzhou City 310003, China

## Abstract

**Background:**

Cancers of digestive system have high case-fatality rate. It is important to find more appropriate methods in diagnosing and predicting gastrointestinal malignances. And thrombospondin-2 (TSP-2) was reported to have the functions, although results were not identical. So we performed this meta-analysis to clarify the significance of TSP-2 in this area.

**Methods:**

PubMed, Embase, Web of Science, Cochrane Library, and Clinicaltrial.gov were searched for relevant studies. Data were extracted from these involved records. For the meta-analysis of diagnostic test, bivariate mixed effect model was used to estimate diagnostic accuracy. For prognosis part, HRs and their 95% CIs were pooled to compare the overall survival (OS) and disease-free survival (DFS) between patients with high TSP-2 and low TSP-2.

**Results:**

Nine records were eligible for the analysis of diagnostic test. Pooled results were as follows: sensitivity 0.60 (0.52, 0.68), specificity 0.96 (0.91, 0.98), positive likelihood ratio (PLR) 15.4 (7.3, 32.2), negative likelihood ratio (NLR) 0.42 (0.34, 0.50), and diagnostic odds ratio (DOR) 37 (18, 76). While in prognosis part, 10 articles were included. Patients with increased TSP-2 had shorter OS (HR = 1.64, 95% CI = 1.21-2.22); however, no difference was found in DFS between TSP-2 high and low groups (HR = 1.44, 95% CI = 0.28-7.33).

**Conclusions:**

TSP-2, as a diagnostic marker, has a high specificity but a moderate sensitivity. Meanwhile, it plays a role in predicting OS. Therefore, making TSP-2 a routine assay could be beneficial to high-risk individuals and patients with digestive malignances.

## 1. Introduction

Cancer is a global health problem [1]. According to the cancer statistics in 2022, around 343,040 persons were newly diagnosed with cancers of digestive system, and 171,920 patients died of it in America. Cancers of the pancreas and liver including intrahepatic bile duct have the lowest 5-year survival rates at only 11% and 20%, respectively [2]. And it was predicted that pancreas cancer would become one of the top cancer killers by 2030 [3]. Therefore, finding effective methods assisting in early diagnosis and accurate monitoring is necessary for better survival.

Thrombospondin (TSP), a matricellular glycoprotein, was first discovered by Baenziger et al. in 1971 [4]. TSP-2 is one of the thrombospondin protein families, which functions by mediating cell-matrix and cell-cell interactions [5, 6]. It is mainly generated by smooth muscle cells and fibroblasts and has an influence on several mammalian biology, such as cell proliferation and migration, tumor angiogenesis, and wound healing [5, 7].

During tumorigenesis, the expression of TSP-2 is changed [8, 9]. Researchers believe that TSP-2 has diagnostic and prognostic value, but conclusions remain inconsistent [10, 11]. Thus, we carried on a meta-analysis assessing the functions of TSP-2, hoping to provide some suggestions for doctors in diagnosing and predicting the cancers.

In this study, the analysis can be divided into diagnostic and prognostic components. In the first part, we assessed the diagnostic accuracy including sensitivity, specificity, likelihood ratio (LR), and area under the curve (AUC). As for the prognosis part, we compared OS and DFS in TSP-2 high and low groups.

## 2. Methods

### 2.1. Search Strategy

PubMed, Embase, Web of Science, Cochrane Library, and Clinicaltrial.gov were searched for relevant studies up to January 20, 2022. The search term included “(Thrombospondin-2) or (TSP-2) or (THBS2)” in Embase, Web of Science, Cochrane Library, and http://Clinicaltrial.gov and MeSH term relating to “Thrombospondin-2” in PubMed. Restricted to the inclusion and exclusion criteria, two reviewers (F.G. and W.C.) screened the records, and discrepancies were reconfirmed by a third reviewer (H.C.).

### 2.2. Selection Criteria

Inclusion criteria for diagnosis part are as follows: (1) patients diagnosed with digestive system cancers, (2) studies assessing diagnostic accuracy of blood TSP-2, and (3) studies with negative (FN). Studies met the following criteria would be included in the prognosis part: (1) clinical studies (both prospective and retrospective), (2) patients included diagnosed with digestive system cancers, (3) studies with comparisons of TSP-2 regarding DFS or OS, and (4) results presented in the form of HR and 95% CI. Exclusions for both parts were as follows: (1) cell studies or animal experiments, (2) replicate studies or studies without full text, and (3) studies lack of interested results or data.

### 2.3. Data Extraction

Data pertaining to author, journal, location, disease, sample size, and cut-off value were extracted. TP, FP, TN, and FN were collected from studies of diagnostic test; HRs and their 95% CIs were from prognosis studies. We used Quality Assessment of Diagnostic Accuracy Studies 2 (QUADAS-2) and Newcastle-Ottawa Scale (NOS) to assess the quality of the records relating to diagnosis and prognosis, respectively.

### 2.4. Statistical Analysis

We used STATA version 16.0 (StataCorp, College Station, TX, USA) for analysis. In the meta-analysis of diagnostic test, specificity, sensitivity, LR, DOR, and AUC were pooled by bivariate mixed effect model. Fagan plots were used to calculate the posttest probability, and Deek's funnel plot was conducted to check the publication bias.

As for the prognosis part, we pooled HRs and their 95% CIs to compare OS and DFS between high TSP-2 and low TSP-2 groups. To explain the relationship of TSP-2 and clinicopathological feature of the patients, OR was calculated. Begg's test and Egger's test were performed to evaluate the publication bias. *I*^2^ statistic and chi-squared test were used to assess the heterogeneity. When *I*^2^ ≤ 50% and *P* > 0.1, the heterogeneity was considered acceptable. Otherwise, random-effects models were employed, and sensitivity and subgroup analyses were performed to account for apparent heterogeneity.

## 3. Results

### 3.1. Study Selection

**Figure 1:**
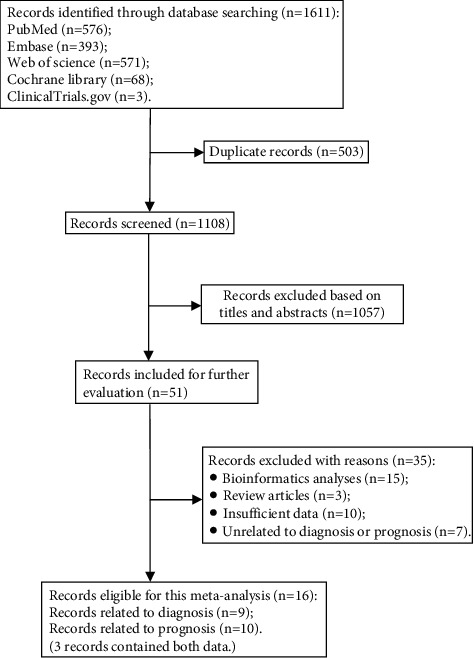
Literature search flow diagram. 1611 articles were screened, and 16 records were finally adopted, 9 relating to diagnosis and 10 relating to prognosis (3 records included both data).

After searching 5 databases mentioned above, a total of 1611 records were identified (Figure 1), of which 503 were duplicates, leaving 1108 for initial screen. 51 were left for further evaluation after screening titles and abstracts. Following detailed reading, 16 relevant studies were included in this meta-analysis. Of these records, 9 are related to diagnosis, and 10 are related to prognosis (3 articles contained both data).

### 3.2. Diagnosis Part

#### 3.2.1. Study Characteristics and Quality Assessment

Main characteristics of 9 included articles were presented in Table 1. A total of 2231 subjects were involved in this meta-analysis of diagnostic test, including 1307 patients diagnosed with digestive cancers and 924 controls. TSP-2 was detected in their blood by enzyme-linked immunosorbent assay (ELISA). Cut-off values varied from 14.85 ng/ml to 42 ng/ml, and two studies did not tell their cut-offs. Results of the quality evaluation according to QUADAS 2 were showed in Supplementary Fig. S1. The risk of bias and the application concerns were at an acceptable level. The Deek's plot was fairly symmetrical, indicating that there was no publication bias either (*P* = 0.53, Supplementary Fig. S2).

#### 3.2.2. Diagnostic Accuracy

**Figure 2:**
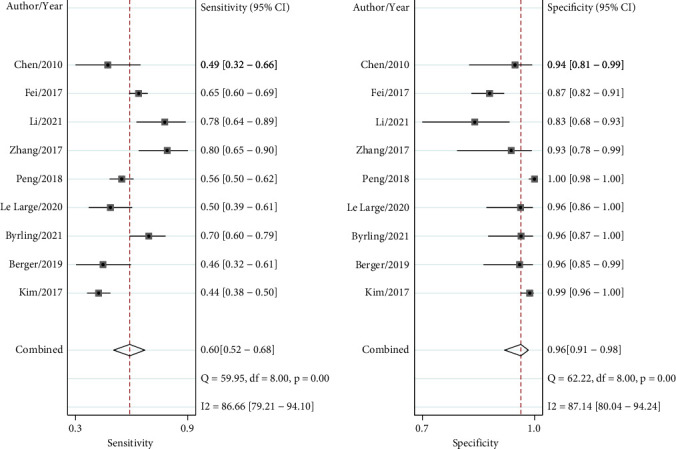
Forest plots of pooled sensitivity and specificity. When thrombospondin-2 (TSP-2) is used for the diagnosis of digestive system cancers, the pooled sensitivity is 0.60 (0.52-0.68), and the specificity reaches as high as 0.96 (0.91-0.98).

**Figure 3:**
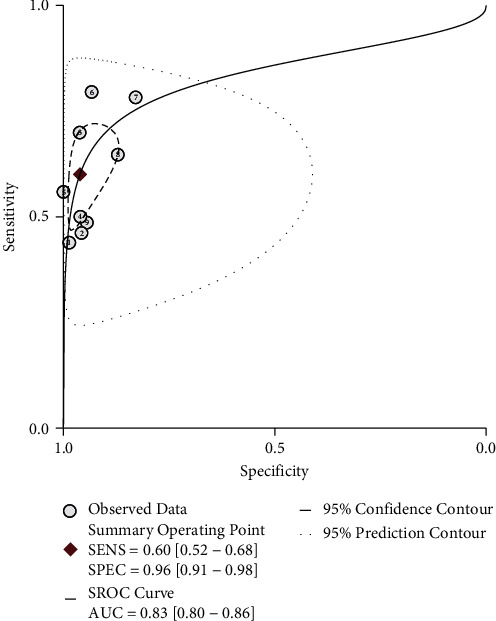
SROC curve of thrombospondin-2 (TSP-2) for the diagnosis of digestive cancers. Each small circle represents the specificity and sensitivity of an included study. The AUC of SROC curve is 0.83. SROC: summary receiver operating characteristics; AUC: area under the curve.

The overall pooled results were as follows: sensitivity 0.60 (0.52, 0.68), specificity 0.96 (0.91, 0.98), PLR 15.4 (7.3, 32.2), NLR 0.42 (0.34, 0.50), and DOR 37 (18, 76). The forest plot of sensitivity and specificity is showed in Figure 2. The AUC of summary receiver operating characteristics (SROC) for TSP-2 was 0.83 (0.80-0.86) (Figure 3). Fagan's plot depicted that if the prior prevalence was 0.5%, the posterior probability would be 7% of PLR and 0.2% of NLR (Supplementary Fig. S3).

### 3.3. Prognosis Part

#### 3.3.1. Study Characteristics and Quality Assessment

Table 2 depicts the characteristics of 10 prognosis studies. There were 1526 individuals involved totally. Six studies tested blood TSP-2, while others detected TSP-2 in tissue. Nine records provided HRs and their 95% CIs regarding OS, while only 3 have the data of DFS. The NOS score of adopted articles was greater than 4 (the total score is 9 points, Supplementary Table S1).

#### 3.3.2. Publication Bias

**Figure 4:**
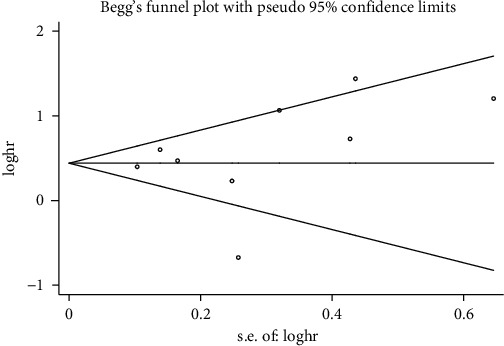
Begg's funnel plot of overall survival (OS). Each point represents an individual study, and points are distributed symmetrically, indicating no publication bias regarding OS.

Nine studies were eligible for Begg's and Egger's tests regarding OS. The results showed that there was no publication bias with regard to OS (Begg's test, *P* = 0.348, Figure 4; Egger's test, *P* = 0.566). Publication bias analysis regarding DFS involved 3 studies and indicated no bias either (Begg's test, *P* = 1.000; Egger's test, *P* = 0.534).

#### 3.3.3. Overall Survival

**Figure 5:**
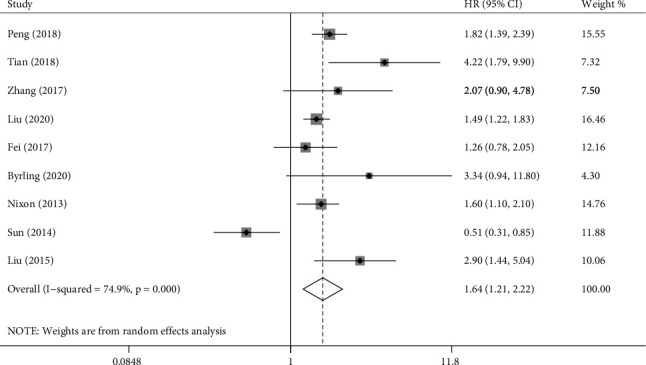
Forest plot comparing the overall survival (OS) between thrombospondin-2 (TSP-2) high and low groups. TSP-2 high group has high risks in OS.

**Figure 6:**
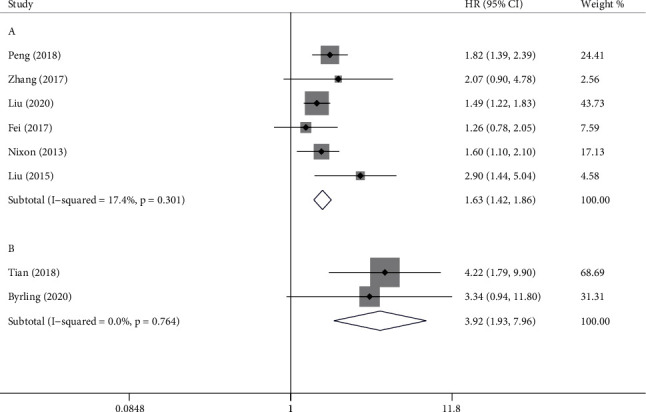
Subgroup analysis of overall survival (OS) according to sample source. (a) Forest plot of thrombospondin-2 (TSP-2) in blood; (b) forest plot of TSP-2 in tissue. TSP-2 has prognostic significance in both subgroups.

Nine studies reported HRs on OS including 1354 individuals. Pooled results demonstrated that the high TSP-2 group had a shorter OS compared with the low TSP-2 group (HR = 1.64, 95% CI = 1.21-2.22, Figure 5). Sensitivity analysis was performed due to significant heterogeneity (*I*^2^ = 74.9%, *P* ≤ 0.001). Outcomes of sensitivity analysis are showed in Table 3. When the study of Sun (2014) was excluded, the heterogeneity became smaller (*I*^2^ = 41.1%, *P* = 0.105). Subgroup analysis was conducted after excluding this study. Heterogeneity becomes less pronounced when studies were divided according to sample size (≤140: *I*^2^ = 0.0, *P* = 0.705; >140: *I*^2^ = 0.0, *P* = 0.530). It was also influenced by sample source (blood: *I*^2^ = 17.4, *P* = 0.301; tissue: *I*^2^ = 0.0, *P* = 0.764, Figure 6). When studies divided by disease type, heterogeneity decreased in pancreatic cancer (*I*^2^ = 0.0%, *P* = 0.546) and hepatobiliary cancer (*I*^2^ = 0.0%, *P* = 0.537), but it increased in colorectal cancer (*I*^2^ = 69.4%, *P* = 0.020). Location and publish year have no apparent effect on heterogeneity (Table 4).

#### 3.3.4. Disease-Free Survival

Data were available in 3 articles, and 275 subjects were analyzed. By pooling the provided HRs of DFS, no significant difference was found between the high TSP-2 and low TSP-2 groups (HR = 1.44, 95% CI = 0.28-7.33). And the forest plot was shown in Supplementary Fig. S4. Further sensitivity analysis and subgroup analysis were not carried on because of the limited number of eligible studies.

#### 3.3.5. Relationship of TSP-2 and Clinicopathological Characteristics

Table 5 describes the associations between TSP-2 and clinicopathological characteristics. TSP-2 was not associated with patients' gender and age (OR = 1.08, *P* = 0.640; OR = 0.91, *P* = 0.702). However, the OR of tumor staging showed that TSP-2 was higher in the stages III-IV than in the stages I-II (OR = 2.26, *P* = 0.002). Neither lymph node metastasis nor vascular invasion was associated with TSP-2 levels (OR = 1.10, *P* = 0.930; OR = 1.31, *P* = 0.843).

## 4. Discussion

As an essential matricellular protein, TSP-2 plays a complicated role in some disease including inflammation, fibrosis, and malignances [12, 13]. It is regarded as a protector in inflammatory diseases. Hou et al. found that TSP-2 could protect cartilage destruction by promoting inflammatory factor in osteoarthritis patients [14]. A study carried on TSP-2 deficient mice, suggesting that TSP-2 could limit inflammatory cell infiltration during delayed-type hypersensitivity [15]. Moreover, TSP-2 can accelerate fibrosis and is considered as a biomarker for liver fibrosis [16–19]. Interestingly, although TSP-2 could hinder angiogenesis, whether it is beneficial or not during tumorigenesis is controversial. Some researchers discovered that TSP-2 could inhibit metastasis of colon cancer and improve survival [20]. Conversely, some studies clarified that TSP-2 promoted cancer growth and invasion in pancreatic cancer [21, 22]. And in intrahepatic cholangiocarcinoma, researchers found elevated levels of TSP-2, inhibiting angiogenesis and promoting lymphangiogenesis, leading to rapid cancer spread [23].

Researchers have already focused on the diagnostic role of TSP-2 in digestive cancers [24]. Kim et al. regarded TSP-2 as an appropriate predictor for early pancreatic ductal adenocarcinoma. TSP-2 combined with CA19-9 could achieve specificity at 98% and sensitivity at 87% [25]. And in the gastric cancer, colorectal cancer, and hepatocellular carcinoma, TSP-2 also aided in diagnosis, especially in combination with other markers [10, 26, 27]. On the other hand, it was reported that TSP-2 was related to survival rates of patients with digestive system cancer, but the results were not identical. In colorectal cancer, some researchers found that patient with high TSP-2 had inferior OS [11]. Some researchers found that the difference in survival between the high and low TSP-2 groups was not statistically significant [10], while others found those with elevated TSP-2 had a longer DFS [28]. And in gastric cancer, OS rates were higher in the high TSP-2 group [29]. The reason may be that TSP-2 can promote the progression of Helicobacter pylori-associated gastric cancer [30].

To our knowledge, this is the first meta-analysis assessing the significance of TSP-2 in diagnosing or predicting gastrointestinal cancers. We found that TSP-2 achieved high overall specificity at 0.96 (0.91, 0.98); however, the sensitivity was moderate. The AUC of 0.83 (0.80, 0.86) for SROC was also indicated a satisfactory diagnosis. Most of the involved papers focused on pancreatic cancer, indicating the importance of TSP-2 in the diagnosis of pancreatic cancer, as was reported in Cancer Discovery [31]. And all of them detect TSP-2 in blood by ELISA. Samples are easy to get without invasion, and the cost is low. Therefore, we regard TSP-2 as a good marker for early screening, especially for those at a high risk of pancreas cancer.

As for the role of TSP-2 in prognosis, the pooled HR indicated that patients with overexpressed TSP-2 had shorter OS. But there was no difference in the DFS rate in the two targeted groups. Subgroup analysis illustrated increased levels of TSP-2 in tissue and blood, and both samples had prognostic value. The relationship with clinicopathological characteristics revealed that TSP-2 increased when tumor staging progressed, which is in consistent with the conclusion TSP-2 increased as cancer developed [21].

How TSP-2 works in gastrointestinal cancers remains unknown. Some researchers believed that inhibition of TSP-2 promoted epithelial mesenchymal transition in gastric cancer, resulting in the progression. But the detailed mechanism was not explained [32]. A study elucidated that TGF-*β*1 could promote the expression of TSP-2 which in turn interacted with integrin *α*v*β*3/CD36, and then activated MAPK signaling to drive the progression of pancreatic cancer [21]. And in colorectal cancer, Xu et al. put forward that TSP-2 accelerated cancer cell proliferation by adjusting the Toll-like receptor 4 (TLR4) [33]. However, there is still a long way to go to unearth the mechanisms of TSP-2 in cancers.

Our study has some limitations. Different samples and cut-offs increased heterogeneity. Some conference abstracts and articles without available full text were not included, leading to publication bias to a certain extent. The number of eligible records involved in our study was not very large. Therefore, articles adopted in each subgroup were limited when subgroup analysis was performed.

## 5. Conclusions

We attach great importance to TSP-2 in the diagnoses and predictions of digestive malignances. TSP-2 alone or in combination with other markers has satisfactory diagnostic accuracy and prognostic value. And TSP-2 in the blood is easily detected, noninvasive, and inexpensive. Therefore, it is strongly recommended to add TSP-2 to the routine detection of tumor markers.

## Figures and Tables

**Table 1:** Characteristics of included studies related to diagnosis.

First author	Year	Journal	Location	Disease	Sample size	Cut-offs (ng/ml)	Patients (*n*)	Controls (*n*)	TP (*n*)	FP (*n*)	FN (*n*)	TN (*n*)
Kim [25]	2017	Science Translational Medicine	USA	Pancreatic cancer	498	42	278	220	122	3	156	217
Berger [34]	2019	Theranostics	Europe	Pancreatic cancer	99	42	52	47	24	2	28	45
Byrling [35]	2021	Clinical and Translational Oncology	Europe	PDAC+dCAA	155	42	103	52	72	2	31	50
Le Large [36]	2020	The Oncologist	Europe	PDAC+dCAA	132	40.9	82	50	41	2	41	48
Peng [37]	2018	Annals of Surgical Oncology	China	Pancreatic cancer	493	29.8	263	230	147	0	116	230
Zhang [27]	2017	British Journal of Cancer	China	Hepatocellular carcinoma	74	36.9	44	30	35	2	9	28
Li [26]	2021	Journal of Oncology	China	Gastric cancer	87	—	46	41	36	7	10	34
Fei [10]	2017	Oncotarget	China	Colorectal cancer	620	14.85	402	218	260	28	142	190
Chen [38]	2010	Pancreas	USA	Pancreatic cancer	73	—	37	36	18	2	19	34

TP: true positive; FP: false positive; FN: false negative; TN: true negative; PDAC: pancreatic ductal adenocarcinoma; dCAA: distal cholangiocarcinoma.

**Table 2:** Characteristics of included studies related to prognosis.

First author	Year	Journal	Location	Disease	Sample size	Sample	OS HR (95% CI)	DFS HR (95% CI)	NOS
Peng [37]	2018	Annals of Surgical Oncology	China	Pancreatic cancer	263	Blood	1.822 (1.389-2.389)	—	6
Lin [28]	2015	American Journal of Translational Research	China	Rectal cancer	172	Tissue	—	0.327 (0.160-0.670)	7
Tian [39]	2018	Official Journal of the Balkan Union of Oncology	China	Colorectal cancer	100	Tissue	4.219 (1.795-9.901)	—	6
Zhang [27]	2017	British Journal of Cancer	China	Hepatocellular carcinoma	44	Blood	2.070 (0.896-4.779)	2.69 (1.203-6.012)	6
Liu [11]	2020	Molecular Cancer Therapeutics	USA	Colorectal cancer	149	Blood	1.49 (1.22-1.83)	—	7
Fei [10]	2017	Oncotarget	China	Colorectal cancer	402	Blood	1.261 (0.776-2.05)	—	6
Byrling [40]	2020	Journal of Translational Medicine	Europe	Cholangiocarcinoma	59	Tissue	3.34 (0.94-11.8)	3.95 (1.09-14.3)	9
Nixon [41]	2013	Clinical Cancer Research	USA	Pancreatic cancer	159	Blood	1.6 (1.1-2.1)	—	7
Sun [29]	2014	Molecular Cancer	China	Gastric cancer	129	Tissue	0.51 (0.31-0.85)	—	8
Liu [42]	2015	Molecular Cancer Therapeutics	USA	Colorectal cancer	49	Blood	2.9 (1.44-5.04)	—	5

NOS: Newcastle-Ottawa scale; OS: overall survival; DFS: disease-free survival.

**Table 3:** Sensitivity analysis of overall survival.

Excluded studies	HR (95% CI)	Heterogeneity	
		*I* ^2^ (%)	*P*
Peng (2018) [37]	1.64 (1.14-2.37)	76.8	≤0.001
Tian (2018) [39]	1.52 (1.13-2.04)	73.6	≤0.001
Zhang (2017) [27]	1.62 (1.17-2.22)	77.8	≤0.001
Liu (2020) [11]	1.72 (1.16-2.55)	77.9	≤0.001
Fei (2017) [10]	1.71 (1.22-2.40)	77.5	≤0.001
Byrling (2020) [40]	1.59 (1.17-2.16)	77.1	≤0.001
Nixon (2013) [41]	1.67 (1.17-2.40)	78.0	≤0.001
Sun (2014) [29]	1.68 (1.47-1.91)	41.1	0.105
Liu (2015) [42]	1.54 (1.13-2.10)	75.0	≤0.001

**Table 4:** Subgroup analysis of overall survival.

	Number of studies	HR (95% CI)	Heterogeneity	
			*I* ^2^ (%)	*P*
Sample size				
≤140	4	2.96 (1.97-4.46)	0.0	0.705
>140	4	1.57 (1.37-1.80)	0.0	0.530
Location				
Europe and America	4	1.61 (1.37-1.90)	42.7	0.155
Asia	4	1.80 (1.45-2.25)	50.0	0.112
Published year				
≤2015	2	1.81 (1.36-2.42)	63.5	0.098
>2015	6	1.64 (1.42-1.90)	43.1	0.118
Sample				
Blood	6	1.63 (1.42-1.86)	17.4	0.301
Tissue	2	3.92 (1.93-7.96)	0.0	0.764
Disease				
Pancreatic cancer	2	1.73 (1.40-2.13)	0.0	0.546
Colorectal cancer	4	1.61 (1.35-1.91)	69.4	0.020
Hepatobiliary cancer	2	2.40 (1.19-4.81)	0.0	0.537

**Table 5:** Associations between TSP-2 and clinicopathological characteristics.

Clinicopathological characteristics	Number of studies	Patient (*n*)	OR (95%CI)	*P*	Heterogeneity	
					*I* ^2^ (%)	*P*
Gender (male vs. female)	5	638	1.08 (0.77-1.52)	0.640	4.7	0.380
Age (older vs. younger)	3	316	0.91 (0.57-1.47)	0.702	0.0	0.614
Tumor staging (III-IV vs. I-II)	3	407	2.26 (1.34-3.82)	0.002	35.6	0.212
Lymph node metastasis (+ vs. -)	3	330	1.10 (0.13-9.30)	0.930	89.7	≤0.001
Vascular invasion (+ vs. -)	3	274	1.31 (0.09-19.00)	0.843	83.1	0.003

## Data Availability

All data generated or analyzed during this study are included in this published article.

## Abbreviations

TSP-2: Thrombospondin-2
OS: Overall survival
DFS: Disease-free survival
PLR: Positive likelihood ratio
NLR: Negative likelihood ratio
DOR: Diagnostic odds ratio
SROC: Summary receiver operating characteristics
LR: Likelihood ratio
AUC: Area under the curve
ELISA: Enzyme-linked immunosorbent assay.

## Supplementary Materials

Supplementary Materials Supplementary Table S1: the Newcastle-Ottawa scale (NOS) quality assessment of the relevant studies. Figure S1: quality of included studies assessed via quality assessment of diagnostic accuracy studies 2. “–” represents high risk/concern; “?” represents unclear risk/concern; “+” represents low risk/concern. Figure S2: Deek's funnel plot evaluating the publication bias. Each circle represents an individual study, and points are distributed symmetrically, representing no publication bias in the meta-analysis of diagnostic test (*P* = 0.53). Figure S3: Fagan plot evaluating the overall diagnostic value of thrombospondin-2 in the diagnosis of digestive cancers. The posterior probability is 7% of PLR and 0.2% of NLR. PLR: positive likelihood ratio; NLR: negative likelihood ratio. Figure S4: forest plot comparing DFS between thrombospondin-2 high and low groups. No difference was found between the two targeted groups. DFS: disease-free survival.
