# Supplementary material for: Diagnostic and Prognostic Roles of Thrombospondin-2 in Digestive System Cancers
Source: Biomed Res Int. 2022 Jul 14;2022:3749306. doi: 10.1155/2022/3749306 (PMC9303135; doi:10.1155/2022/3749306)
Supplement: Supplementary Materials — Supplementary Table S1: the Newcastle-Ottawa scale (NOS) quality assessment of the relevant studies. Figure S1: quality of included studies assessed via quality assessment of diagnostic accuracy studies 2. “–” represents high risk/concern; “?” represents unclear risk/concern; “+” represents low risk/concern. Figure S2: Deek's funnel plot evaluating the publication bias. Each circle represents an individual study, and points are distributed symmetrically, representing no publication bias in the meta-analysis of diagnostic test (P = 0.53). Figure S3: Fagan plot evaluating the overall diagnostic value of thrombospondin-2 in the diagnosis of digestive cancers. The posterior probability is 7% of PLR and 0.2% of NLR. PLR: positive likelihood ratio; NLR: negative likelihood ratio. Figure S4: forest plot comparing DFS between thrombospondin-2 high and low groups. No difference was found between the two targeted groups. DFS: disease-free survival. [file 3749306.f1.doc]

**Supplementary materials**

**Supplementary Table S1 The newcastle-ottawa scale (NOS) quality assessment of the relevant studies.**

|  | **Selection** | | | |  | **Outcome** | | |  |
| --- | --- | --- | --- | --- | --- | --- | --- | --- | --- |
| **Study** | **Exposed cohort** | **Nonexposed cohort** | **Ascertainment of exposure** | **Outcome of interest** | **Comparability** | **Assessment of outcome** | **Length of follow-up** | **Adequacy of follow-up** | **Total score** |
| Peng (2018) | ★ | ★ | ★ | ★ | ☆ | ☆ | ★ | ★ | 6 |
| Lin (2015) | ★ | ★ | ★ | ★ | ☆ | ★ | ★ | ★ | 7 |
| Tian (2018) | ★ | ★ | ★ | ★ | ☆ | ☆ | ★ | ★ | 6 |
| Zhang (2017) | ★ | ★ | ★ | ★ | ☆ | ☆ | ★ | ★ | 6 |
| Liu (2020) | ★ | ★ | ☆ | ★ | ★ | ★ | ☆ | ★ | 7 |
| Fei (2017) | ★ | ★ | ★ | ★ | ☆ | ☆ | ★ | ★ | 6 |
| Byrling (2020) | ★ | ★ | ★ | ★ | ★ | ★ | ★ | ★ | 9 |
| Nixon (2013) | ★ | ★ | ☆ | ★ | ★ | ★ | ☆ | ★ | 7 |
| Sun (2014) | ★ | ★ | ★ | ★ | ★ | ☆ | ★ | ★ | 8 |
| Liu (2015) | ★ | ★ | ★ | ★ | ☆ | ☆ | ☆ | ★ | 5 |
| ★: A score is given; ☆: Zero score is given. | | | | | | | | | |

**
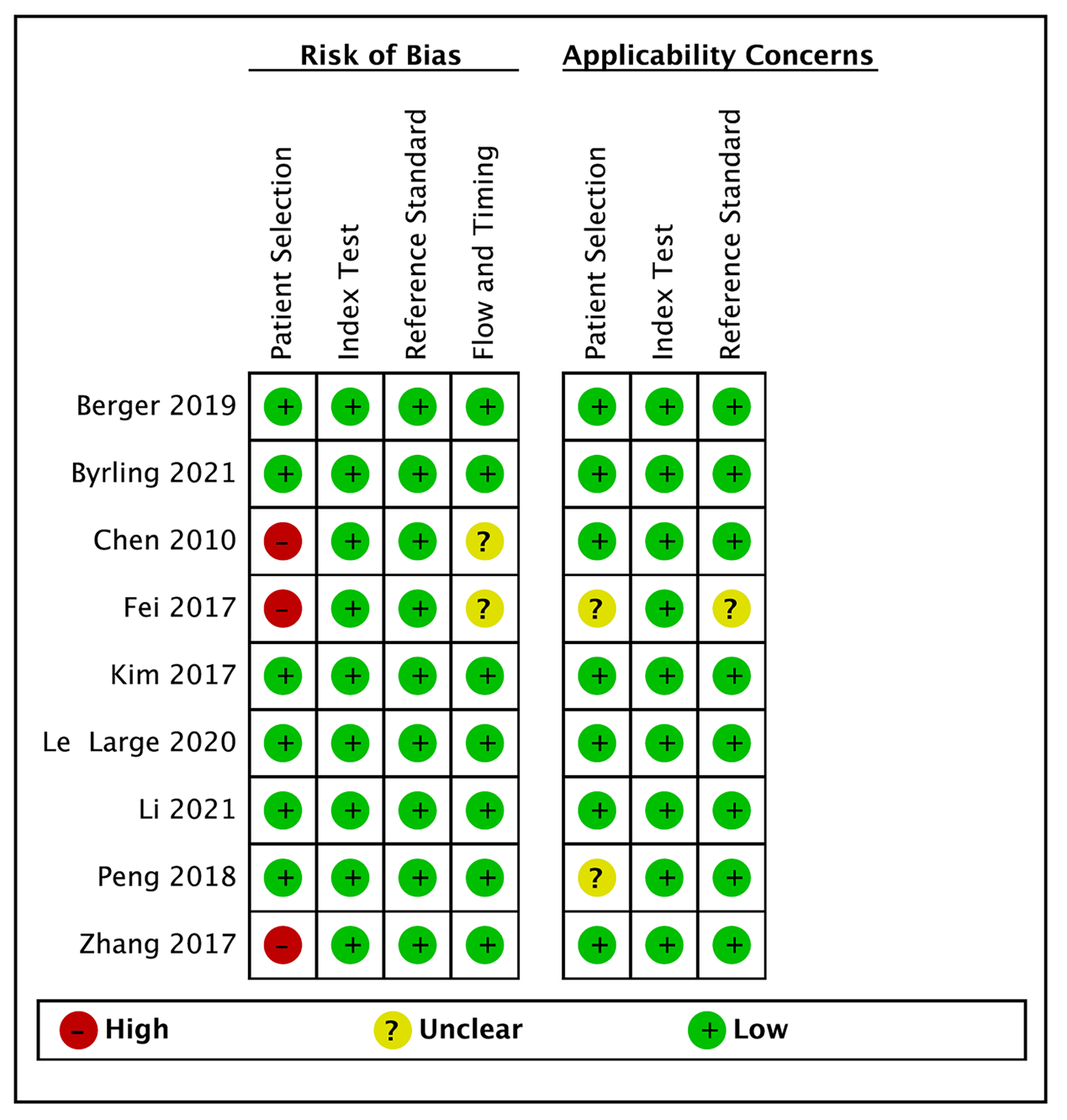
**

**Fig. S1 Quality of included studies assessed via quality assessment of diagnostic accuracy studies 2.** “–” represents high risk/concern; “?” represents unclear risk/concern; “+” represents low risk/concern.

**
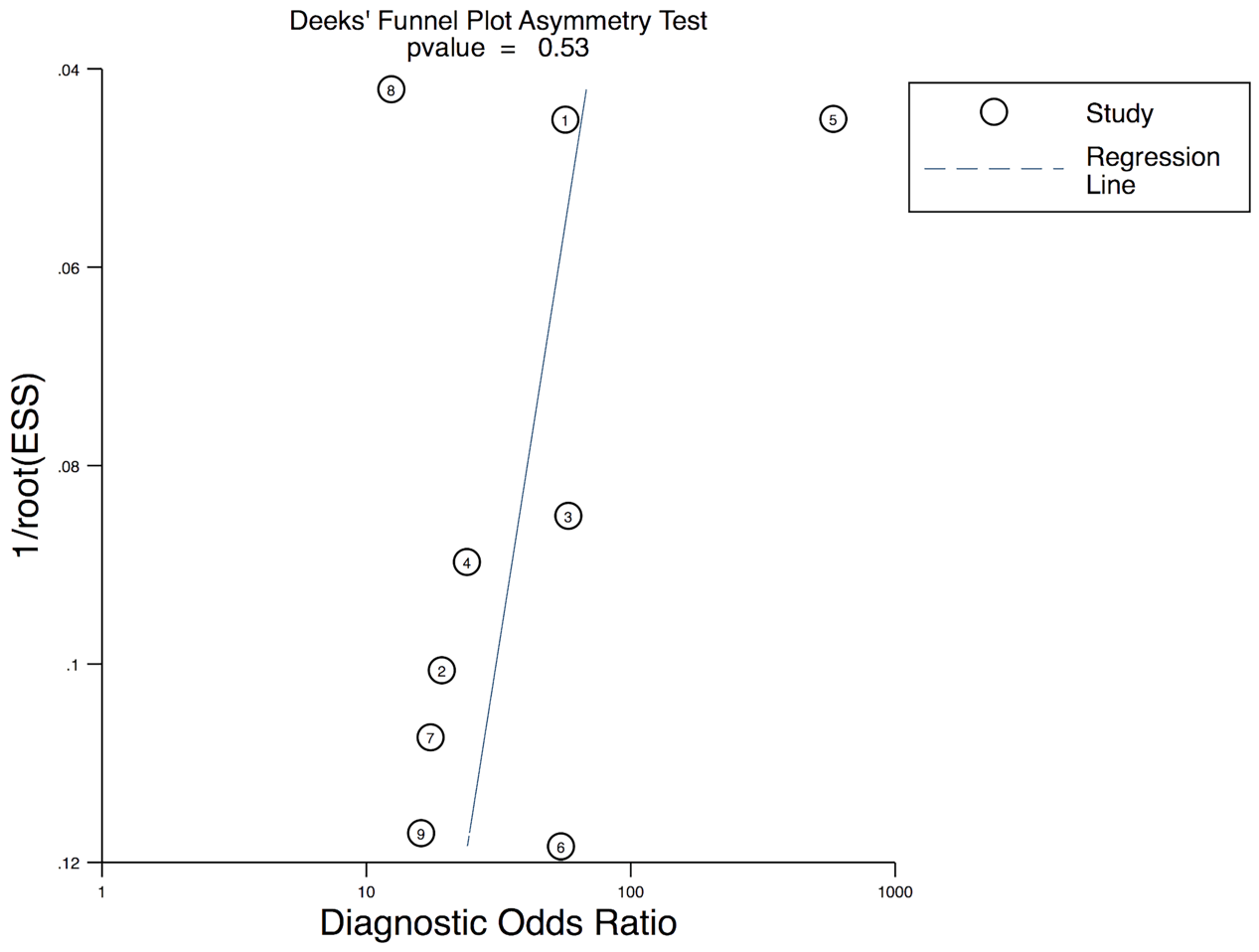
**

**Fig. S2 Deek’s funnel plot evaluating the publication bias.** Each circle represents an individual study, and points are distributed symmetrically, representing no publication bias in the meta-analysis of diagnostic test (*P*=0.53).

**
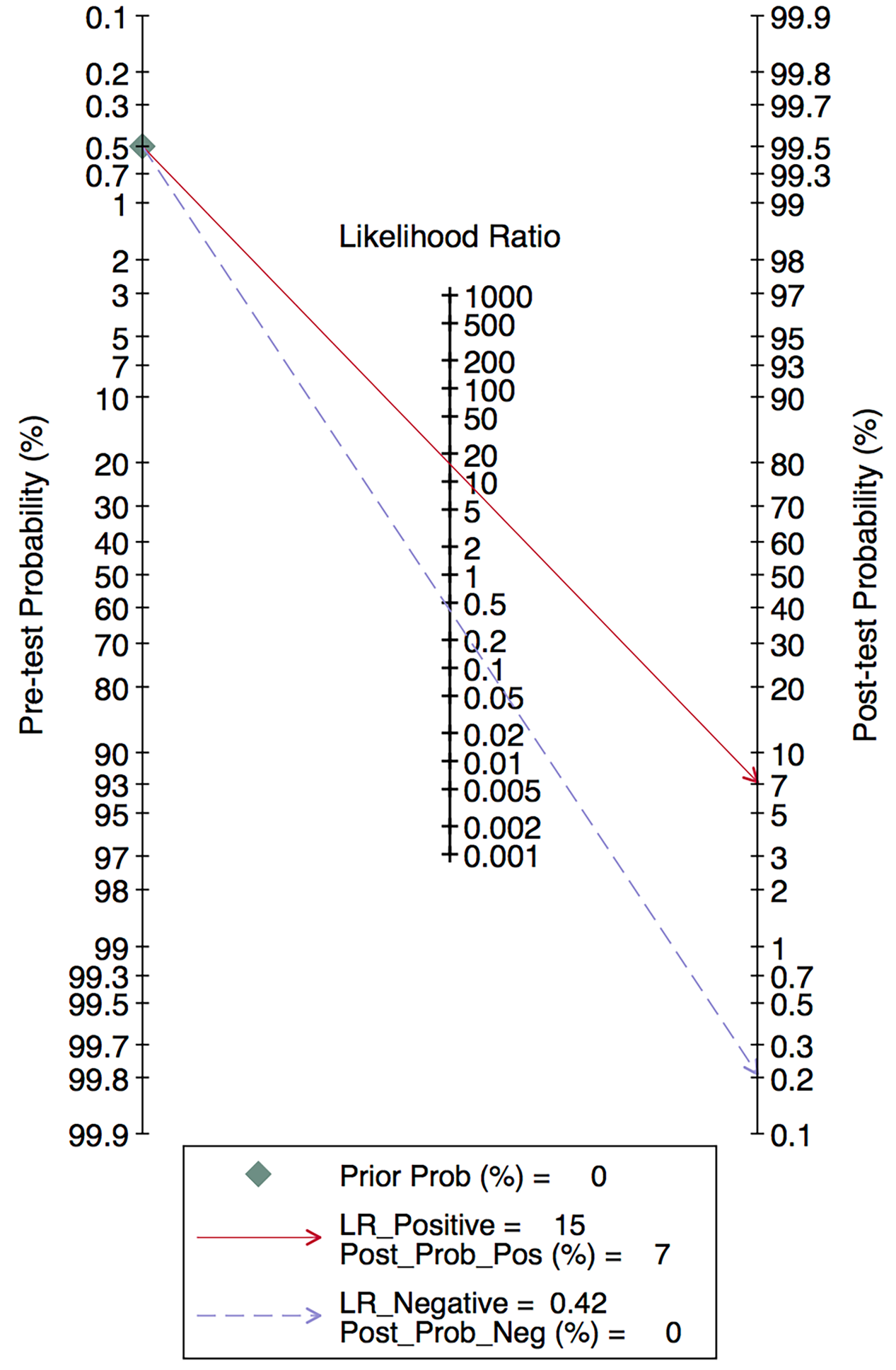
**

**Fig. S3 Fagan plot evaluating the overall diagnostic value of thrombospondin-2 in the diagnosis of digestive cancers.** The posterior probability is 7% of PLR and 0.2% of NLR. PLR: positive likelihood ratio; NLR: negative likelihood ratio.

**
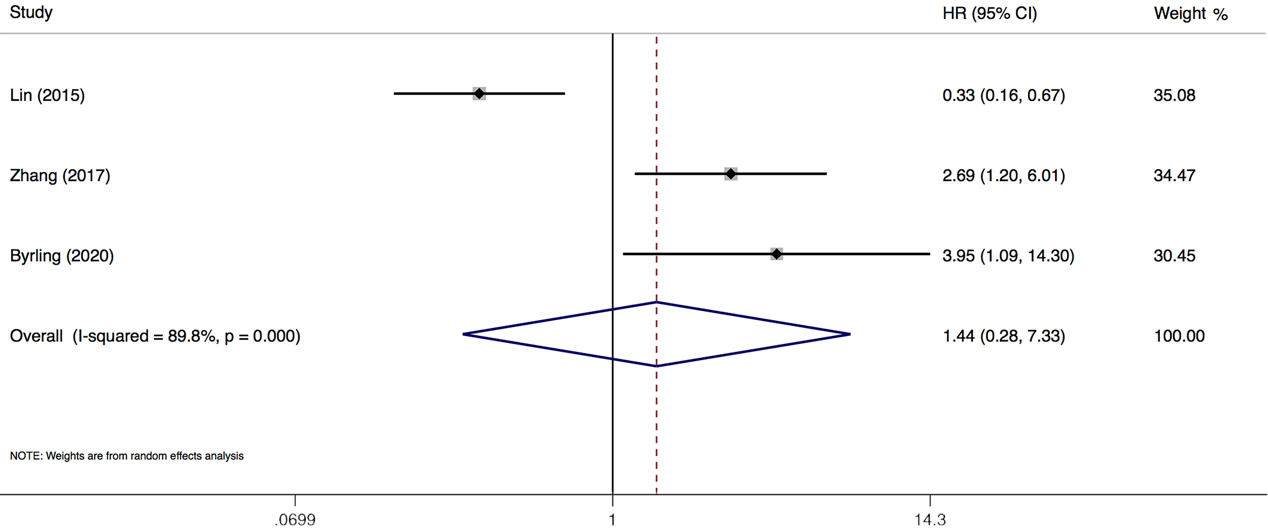
**

**Fig. S4 Forest plot comparing DFS between thrombospondin-2 high and low groups.** No difference was found between the two targeted groups. DFS: disease-free survival.
